# Ablation of Ventricular and Atrial Arrhythmias in the Era of Cardiac Magnetic Resonance

**DOI:** 10.1111/echo.70150

**Published:** 2025-04-07

**Authors:** Pietro Costantini, Francesca Coraducci, Giulia De Zan, Damiano Fedele, Eleonora Ostillio, Riccardo Bertozzi, Federico Donato, Giuseppe Muscogiuri, Anna Giulia Pavon, Luca Bergamaschi, Carmine Pizzi, Astrid Hendriks, Anneline S. J. M. te Riele, Dominika Suchá, Sophie Rier, Pim van der Harst, Birgitta Velthuis, Ivo van der Bilt, Anna Colarieti, Alessandro Carriero, Marco Guglielmo

**Affiliations:** ^1^ Radiology Department Azienda Ospedaliera Maggiore della Caritá di Novara University of Eastern Piedmont Novara Italy; ^2^ Department of Biomedical Sciences and Public Health Marche Polytechnic University Ancona Italy; ^3^ Division Heart and Lung Cardiology Department University Medical Center Utrecht Utrecht The Netherlands; ^4^ Department of Medical and Surgical Sciences (DIMEC) Alma Mater Studiorum University of Bologna Bologna Italy; ^5^ Cardiovascular Division Morgagni‐Pierantoni University Hospital Forlì Italy; ^6^ Department of Diagnostic and Interventional Radiology Papa Giovanni XXIII Hospital Bergamo Italy; ^7^ Division of Cardiology Cardiocentro Ticino Institute Ente Ospedaliero Cantonale Lugano Switzerland; ^8^ Faculty of Biomedical Sciences Università Della Svizzera Italiana Lugano Switzerland; ^9^ Department of Radiology University Medical Center Utrecht Utrecht The Netherlands; ^10^ Department of Cardiology Haga Teaching Hospital The Hague Netherlands

**Keywords:** atrial ablation, late gadolinium enhancement, magnetic resonance imaging, post‐ablation, pulsed field ablation, real‐time ablation, ventricular ablation

## Abstract

In the past decade, cardiac magnetic resonance (CMR) has undergone remarkable progress, emerging as a pivotal tool in various cardiological scenarios. Its capacity for tissue characterization, both with and without contrast agents, makes CMR the perfect tool to study the substrate of arrhythmia. This review highlights the potential role of CMR in electrophysiology (EP) and its role in the ablation of atrial and ventricular arrhythmias. First, we will discuss the key aspects of ventricular arrhythmia ablation, while in the second part, we will review how CMR is changing the ablation of atrial arrhythmias. The potentiality of CMR in the pre‐procedural, intra‐procedural, and post‐ablation assessment will be reviewed. In particular, CMR is capable of visualizing fibrosis and building 3D reconstruction. Furthermore, it is possible to merge a 3D‐rendered shell of the heart into the EP room to guide radiation‐free ablation through active or passive tracking. Finally, the accuracy of CMR in depicting ablation lesions and its ability to predict arrhythmia relapses will be discussed.

## Graphical abstract

Cardiac magnetic resonance (CMR) has significantly advanced over recent decades, becoming indispensable in various settings. In electrophysiology (EP), CMR plays a pivotal role in procedural planning, real‐time guidance, and post‐procedural evaluation. Its precise definition of myocardial scarring tailor's ablation processes, while the possibility of crafting 3D models of the heart and merging them into the EP lab is emerging. Additionally, specialized sequences assess ablation outcomes and predict arrhythmia recurrence, highlighting its prognostic value.

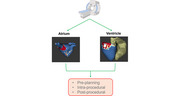

**Abbreviations**
AF: atrial fibrillation
AT: active tracking
CMR: cardiac magnetic resonance
EAM: electro‐anatomical map
ECG: electrocardiogram
ECV: extracellular volume
EP: electrophysiology
FWHM: full width at half maximum
HTC: heterogeneous tissues channel
ICD: implantable cardiac defibrillator
IIR: image intensity ratio
LA: left atrium
LAVA: local abnormal ventricular activity
LGE: late gadolinium enhancement
MVO: microvascular obstruction
PFA: pulsed field ablation
PT: passive tracking
PVI: pulmonary vein isolation
RF: radiofrequency
SD: standard deviations
TI: time of inversion
VA: ventricular arrhythmias
VT: ventricular tachycardia

## 1 Introduction

Treating cardiac arrhythmias is a major challenge for physicians worldwide and it currently relies on different strategies, from pharmacological therapy to implantable cardiac devices and catheter ablation. Lately, great advancements have been made in catheter ablation therapy, especially in terms of equipment and mapping techniques that serve as a roadmap for the procedure. Nevertheless, unsuccessful ablation is not rare, with consequences on patient's quality of life and morbidity, especially in ventricular tachycardia (VT) ablations. The greatest challenges related to VT ablation consist in recognizing and reaching the anatomical area representing the substrate of the arrhythmia, as well as in understanding the success of the procedure in terms of arrhythmia recurrence. Indeed, in certain patients, an endocardial mapping could miss an epicardial focus of arrhythmia or vice versa. Moreover, acute myocardial tissue alteration might reduce electrical conductivity among cells and mimic a successful ablation [1].

In recent decades, cardiac magnetic resonance (CMR) has made significant advancements in the realm of electrophysiology (EP). Today, a plethora of applications leveraging CMR have emerged to refine the process of ablation. In this comprehensive review, we endeavor to elucidate the evidence and address the uncertainties surrounding the utilization of CMR across various facets of ablation procedures.

## 2 The Role of CMR in Ventricular Arrhythmias

Ventricular arrhythmias (VA) are one of the leading causes of sudden cardiac death [2]. Among these, VT is a life‐threatening arrhythmia that often arises from an abnormal focus or electrical circuit in the ventricular myocardium. VT has a broad and diverse etiology, ranging from structural heart disease to nonstructural heart disease. CMR can detect this abnormal structural substrate, such as those seen in ischemic heart disease, post‐myocarditis, and cardiomyopathies [3].

Ischemic injuries and late‐stage cardiomyopathies are associated with myocardial fibrosis. Fibrotic changes promote a substrate where re‐entry can occur and emerge triggers to interact in the development of VTs [4].

The first‐line therapy for patients with VT involves medical treatment to reduce the arrhythmic burden and the use of an implantable cardiac defibrillator (ICD) to prevent sudden cardiac death. Although ICDs are effective at preventing fatal outcomes, they do not eliminate the occurrence of VTs [2]. Furthermore, pharmacological treatments alone are often insufficient to control VT [5]. Frequent ICD shocks in patients with VAs can significantly decrease quality of life and are associated with higher rates of hospitalization and mortality. [3]. Consequently, radio frequency (RF) ablation represents an effective non‐drug therapy against VA [6]. CMR represents a valid technology for managing every step of the procedure.

### 2.1 Planning

A tailored planning approach is vital for ventricular ablation. A conventional 12‐lead electrocardiogram (ECG) can already give important information about the localization of the exit of the arrhythmia, including whether it is endocardial or epicardial. However, its sensitivity and specificity are moderate and extremely variable from case to case, mainly due to extensive fibrosis and the use of anti‐arrhythmic drugs [7, 8]. Gaining information on the exact location of the arrhythmic substrate in the ventricle is, however, crucial to guide procedural planning (e.g., whether an epicardial or endocardial approach is warranted) of the ablation, which may simultaneously shorten the procedural time.

Consequently, the most common approach is to outline the re‐entry circuits by reconstructing an electro‐anatomical map (EAM) of the ventricles in the EP lab through dedicated software. Mapping the re‐entry circuit requires VT induction. However, many patients render non‐inducible throughout a VT ablation procedure. In addition, patients are exposed to a risk of hemodynamic instability. During sinus rhythm, substrate mapping can be performed by identifying the areas of slow conduction. Yet, identifying all late potentials and local abnormal ventricular activity (LAVA) may be time‐consuming.

On the contrary, scars responsible for the re‐entry circuit can be easily visualized on CMR with different methods. The most utilized technique is late gadolinium enhancement (LGE) visualization after gadolinium injection, a contrast agent that spreads into fibrotic tissues due to extracellular volume (ECV) expansion. Conventional LGE imaging consists of T1‐weighted sequences acquired 10–20 min after 0.15–0.2 mmol/kg gadolinium‐based contrast agent administration [9]. To avoid artifacts, sequential breath‐holds are required to the patient. The most used 2D sequences are the so‐called “bright blood” sequences where myocardium appears hypointense, and blood appears hyperintense. Novel sequences such as dark blood and flow‐independent dark blood delayed enhancement (FIDDLE) have been developed. They provide better contrast signal between blood and enhanced myocardium and help to perceive subtle subendocardial scars [10, 11]. However, studies utilizing the latter sequences for VA ablation are lacking.

An initial study by Andreu et al. evaluated the clinical significance of analyzing scarred ventricular tissue to determine the VA site and guide the appropriate ablation strategy [12]. The study underlined the importance of a pre‐ablation assessment of cardiac LGE, in terms of presence/absence and of its localization (subendocardial, mid‐myocardial, epicardial, and transmural). The presence of subepicardial LGE yielded a sensitivity of 84.5% and a specificity of 100% in predicting an epicardial origin of VA.

Subsequently, Malaczynska‐Rajpold et al. compared post‐infarct patients presenting either with sustained VT (case group) or without sustained VT (control group) [13]. The heterogeneous zone was visualized on 2D sequences LGE and was defined as scar tissue with signal density lower than normal scar (between two and three times the normal myocardium intensity) [14]. Islets of heterogeneity were significantly higher in the case group compared to the control group suggesting an association between islets of LGE heterogeneity and conducting channels [13]. Conducting channels refer to areas characterized by the presence of a slow conduction zone simultaneously surrounded by scar and connected to a healthy myocardium, that establishes the electrical substrate for re‐entry [15].

**FIGURE 1:**
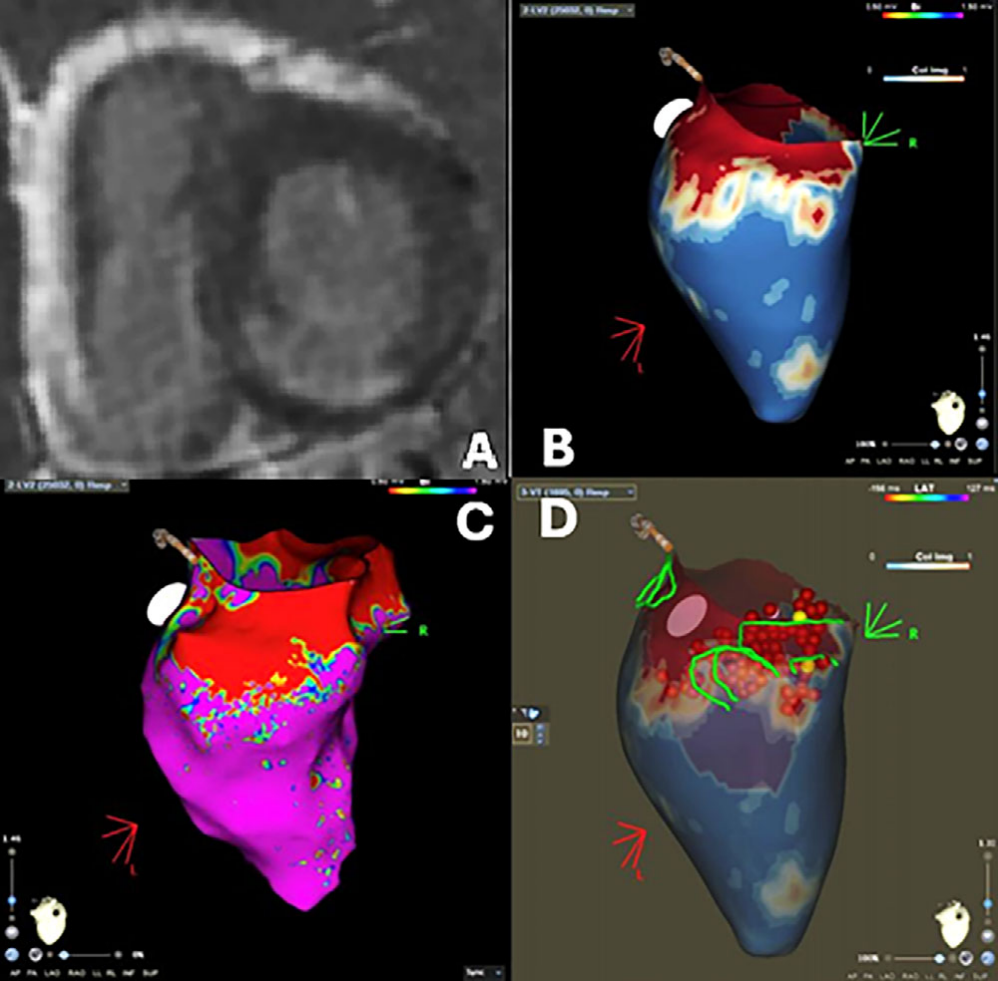
Acquisition and post‐processing of an LV fibrotic area. (A) Short‐axis LGE sequence showing a transmural (>50%) enhancement of the basal infero‐septal wall and basal inferior wall. (B) 3D reconstruction with dedicated software (ADAS 3D, La Galgo Medical, Barcelona, Spain). The colorimetric map is based on signal intensity. The map can be used to mark the islets of heterogeneity. (C) Corresponding endocardial voltage map (Carto, Biosense Webster) indicating the discrete area of low voltage (red area) in the LV. (D) Mid myocardial conducting channels as found with the 3D reconstruction of CMR images merged in the EP laboratory in which the red dots represent extensive RF ablation.

In recent years, novel CMR techniques have emerged. New 3D free‐breathing LGE sequences led to higher spatial resolution allowing better reconstruction of the myocardium increasing the capability to evaluate its signal intensity. Multiple software has been designed to process 3D LGE information and define the extension and presence of conducting channels and have proved to be better than conventional 2D LGE sequences (Figure 1) [16, 17].

T1 mapping is also a valid option to assess fibrotic changes. T1 values increase along with the increase of fibrotic or watery content in the myocardium [18]. T1 mapping requires multiple image acquisitions with different T1 weightings in order to fit the signal intensities of the images into an equation. By doing so, the reader obtains a quantitative map that represents tissue characterization from which region of interest can be drawn to assess tissue changes. T1 mapping sequences have been proven to be more sensible for early or diffuse fibrosis compared to LGE, especially for non‐ischemic cardiomyopathy [18]. After 10–15 min from contrast injection, post‐contrast T1 mapping can be acquired. ECV can be estimated through the measurement of myocardial and blood T1 before and after contrast administration and the patient's hematocrit [18]. ECV is strictly correlated with variations in the inter‐myocyte space. It tends to increase because of fibrotic and edematous deposition in this space. That being said, local or diffuse increases in ECV values can indicate a potential VT substrate, although in clinical practice, the identification of LGE still remains the mainstay of tissue characterization. In a retrospective study on patients without LGE that underwent VT ablation, T1 post‐contrast value correlated with VT recurrence after ablation and the burden of diffuse fibrosis measured via T1 post contrast showed to be associated with the extent of the abnormal electrical substrate on EAM [19].

### 2.2 Intra‐Procedural

#### 2.2.1 LGE CMR Integration

At present, the vast majority of VT ablations are guided by EAMs. By registering the electrical characteristics of the mapped area, the anatomy of the heart is reconstructed and the conducting channels may be identified, thus reducing fluoroscopy times.

**FIGURE 2:**
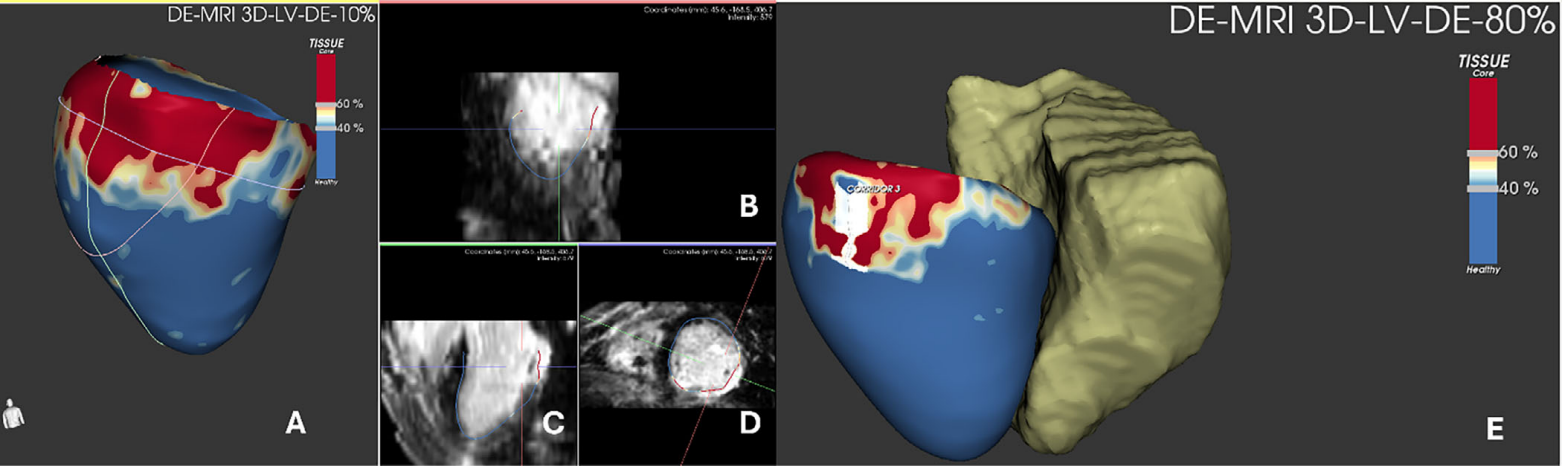
Post‐processing of a LV fibrotic area of the inferolateral wall. (B–D) Multiplanar assessment of LGE via 3D LGE sequence. The post‐processing comprehends a first phase of segmentation followed by a 3D reconstruction based on signal intensity (A). (E) The dedicated software (ADAS 3D, La Galgo Medical, Barcelona, Spain) allows for the integration of another cardiac structure (in this case the right ventricle) from cardiac computed tomography.

In a non‐randomized, consecutive study, Andreu et al. merged a 3D CMR map with EAM in patients undergoing ablation due to sustained monomorphic VT. A pre‐ablation CMR was acquired for every patient to create a 5‐layer‐shell of the left ventricle thickness. Then, pixel signal intensity (PSI) maps were derived and interpolated into the layers so that the delineation of scar areas and border zones was based on intensity thresholds and heterogeneous tissues channel (HTC) was defined as a continuous line, “corridor”, of border zone surrounded by scar tissue that connects two healthy myocardium zone on LGE 3D CMR reconstruction. The resulting 3D shell of the heart with scar and HTC identification was then integrated with the EAM to perform the VT ablation (Figure 2). Patients in the case group (where a CMR‐aided VT ablation was performed) showed a significantly lower rate of VT inducibility after substrate ablation and higher rate of VA‐free survival. In contrast to CMR‐guided VT ablation, fluoroscopy times were not different between CMR‐aided and EAM‐only VT ablation procedures, and a possible explanation resides in the fact that CMR‐aided VT ablation still requires an EAM to be performed as CMR could not be the only guidance for the procedure [20]. Similar results have been reported by multiple studies, whether the ablation was conducted with EAM integration or solely based on 3D‐LGE CMR [20, 21, 22]. Moreover, a higher recurrence rate was observed even in patients who underwent previous CMR but with no interpolation during ablation, thus underlying the power of CMR‐EAM integration [23].

#### 2.2.2 Real‐Time Visualization

Besides the use of previously acquired LGE images to integrate the EAM with anatomical data, CMR imaging can also be used in real‐time during the procedure to guide the catheters to the target zone. In this case, the procedure takes place inside the CMR suite, with the patient lying inside the bore of the CMR scanner. Two techniques can be performed for real‐time catheter visualization: passive tracking (PT) and active tracking (AT). In PT the catheter is depicted thanks to the artifact caused by ferromagnetic materials at the tip of the catheter, thus creating a signal void in T2‐weighted images. The major drawbacks of the PT are the limited spatial (especially in the moving heart) and temporal resolution (usually the frame rate is 2–8/s) [24].

AT addresses these problems by implementing coils into the catheter tip that sends a receiver signal. Catheter motion is therefore tracked by dedicated CMR tracking sequences, thus allowing a real‐time integration with a 3D reconstruction of the heart with an impressive accuracy (0.9 ± 0.6 mm) [25]. The 3D reconstruction of the heart is based on a pre‐acquired 3D data set acting as “roadmap” [26]. The major issue of AT is the need for a perfect alignment between the pre‐intervention 3D reconstruction and the AT tracking sequence as the catheter tip is visualized based on CMR coordinates. Thus, insufficient body alignment between the pre‐ and intra‐operative position and respiratory motion can lead to misalignment that requires correction by repetition of the 3D CMR [27]. In addition, not only interventional equipment but also all monitoring equipment (e.g., electrocardiograms and oxygen saturation monitors) must be compatible with the magnetic resonance imaging (MRI) environment. Moreover, electrophysiologists, nurses, and technicians must undergo special training regarding this aspect. One of the most serious complications linked to ventricular ablation is ventricular fibrillation or pulseless VT, which must be treated immediately by cardioversion. At the moment, there are no commercially available MRI‐compatible external defibrillators [28]. Very few studies focused on external defibrillators in the MRI room and are either animal ones or with a customized defibrillator [29, 30]. In addition, most patients undergoing ablation have ICDs possibly creating further ferromagnetic artifact to the procedure.

For these reasons, until now real‐time ventricular ablation has been performed only in animal studies and not yet performed on human patients [31].

### 2.3 Post‐Ablation

The recurrence rate after VT ablation is still high (30%–40%) and it is often linked to the original re‐entry circuit or LAVA areas [32, 33]. In this regard, it is believed that RF‐induced lesion visualization can help predict arrhythmia recurrence. Lesion assessment using CMR after RF catheter ablation of VT can reveal the presence of edema, enhanced myocardium, and microvascular obstruction (MVO) after ablation [1, 34–36].

All these typical RF‐induced alterations can be visualized through T1‐weighted, T2‐weighted, and LGE sequences. RF delivery induces coagulative necrosis and edema, which both lead to ECV expansion. However, the relationship between ECV and LGE changes is more complex than this statement suggests. Due to the transient nature of these changes, lesion can be overestimated using LGE or T2‐weighted sequences and may not fully reflect the underlying tissue remodeling [37].

Several studies focused on native T1‐weighted images for lesion visualization. In an animal study on swine, pre‐ and post‐ablation CMRs were compared to pathology. Native T1‐weighted images had a good correlation with the necrotic core of the lesion [38]. Kholomovski et al. analyzed acute and chronic left ventricular injuries in a canine model showing how acute T1‐weighted lesion size showed a good correlation with chronic histological lesion while LGE appeared to overestimate it. No chronic lesion was observed on long time of inversion (TI) T1‐weighted images thus resulting in a good predictor of chronic lesion size and a great distinguisher between acute or chronic lesions [39].

Krann et al. compared native and post‐contrast sequences for the visualization of real‐time lesions during an AT VT ablation in eight swine [33]. A core hypothesis of this study is that transient edema alters ECV and cell‐to‐cell coupling thus reducing electrical conduction and giving a misleading impression of successful ablation. The T2‐weighted images, depicting edema, showed a larger lesion compared to the native T1‐weighted depiction of the RF lesion, while bipolar voltages could not appreciate the differences. As edema tends to reduce over time, this could explain the late recurrences after ablations [1, 34].

Multiple studies have confirmed a significant lesion size shrinkage from the acute to chronic phase, appreciable in T2‐weighted and LGE sequences, and a nearly complete regression of MVO [34, 40]. A recent study by Roca‐Luque et al. compared the appearance of chronic ablated lesions in LGE CMR with pre‐intervention CMR in patients who underwent first‐procedure VT ablation **[** 36 **]**. In both CMRs, an LGE‐based 3D volume rendering was performed through semi‐automated segmentation on short axis with dedicated software. For both pre‐ and post‐ablation CMRs, conducting channels were counted and their differences were compared to the clinical outcome obtained after ablation. The difference in terms of conducting channels correlated with the recurrence rate (a reduction of conducting channels <55% yielded a sensibility of 100% and a specificity of 61% to predict VT recurrence). Moreover, this study enlightens the non‐significance of MVO presence in the acute phase as a marker for successful ablation [36]. This being said, enhanced sequences appear to be more suitable for pre‐ and post‐procedural chronic visualization, while their peri‐procedural usefulness is debatable due to the edema component.

All the previous studies focused on RF‐induced lesions. Newer technologies such as pulsed field ablation (PFA) are still not approved for human VT ablation, but good results on animals and a few case reports on human subjects have been published [41]. PFA technology induces cellular apoptosis by disrupting myocyte cellular membrane with electrical pulses, thus reducing ECV expansion. To the best of our knowledge, no studies have been done focusing on the imaging assessment of ventricular ablation lesion performed using PFA.

## 3 The Role of CMR in Atrial Arrhythmias

Atrial fibrillation (AF) is the most common arrhythmia and appears as a progressive disease that causes atrial remodeling, which, in turns, leads to an increased occurrence of AF (the so‐called “AF begets AF”) [42]. As widely recognized for other arrhythmias, fibrosis has a pivotal role in a structural remodeling and is directly involved in the onset and perpetuation of focal and re‐entry mechanisms of atrial arrhythmias [43]. Different studies described a strong correlation between the fibrotic burden and the worsening of atrial function [44, 45].

### 3.1 Planning

Before atrial flutter or AF procedures, CMR can be used to study the anatomy of the left atrium and pulmonary veins and to rule out left atrial (LA) appendage thrombus. Moreover, CMR can be utilized to characterize the tissue of the left atrium.

Multiple studies have linked LGE and areas of low voltage in the EAM in patients with AF [46]. Additionally, LGE has been demonstrated to be more extensive in patients with persistent AF and those with greater LA remodeling, as opposed to patients with paroxysmal AF [44, 47]. Moreover, regardless of AF persistence at baseline, LA LGE extension >35% was found to be an independent factor for AF recurrence post‐ablation [44].

Recently, new 3D‐LGE sequences have been developed. They allow the imaging of the whole heart with high signal‐to‐noise and contrast‐to‐noise ratio and without slice gaps, enabling reformatted images in any desired plane.

The first developed sequences required multiple long (>20 s) breath‐holds, making their application difficult on vulnerable patients. To overcome this issue, new free‐breathing sequences are now used, combining phase‐sensitive inversion recovery sequences with submillimeter voxel size and targeting diaphragm motion for the acquisition [48]. Despite the clear evidence that links fibrosis to AF onset and severity, there is no clear consensus on how to quantify LA fibrotic burden. Although picturing ventricular scar, the most common methods are based on signal intensity above normal myocardium, with 2‐, 3‐, 4‐, 5‐, or 6‐standard deviations (SD) above the remote myocardium or the full width at half maximum (FWHM) technique [49]. The FWHM considers the threshold value as half of the maximal signal within the infarcted area [50]. To the best of our knowledge, there is no widespread consensus on whether SD‐based or FWHM‐based quantification is more suited for LA LGE. The FWHM is among the most reliable methods but yields intrinsic limitation when encountering homogeneous lesions [50]. A target lesion with a signal intensity 2 SD above the mean is generally considered statistically significant. That said, there is ongoing debate regarding the optimal SD gradient to use. Hwang et al. studied thirty‐eight patients undergoing atrial ablation, comparing different SD thresholds to determine the most accurate one. Among the thresholds tested — 2‐SD, 3‐SD, 4‐SD, 5‐SD, and 6‐SD — the 6‐SD proved to be the most accurate. Moreover, FWHM was also evaluated, and together with the 6‐SD threshold, it was found to be statistically reproducible, with an intraclass correlation coefficient greater than 0.7 [51]. In recent years, new methods involving intensity thresholds were proposed to allow for objective scar recognition and interpatient comparisons.

Khurram et al. proposed and validated a normalized measure, the image intensity ratio (IIR), for the assessment of scars. The IIR was calculated by dividing the myocardial signal intensity by the mean LA blood pool intensity. Higher local IIR thresholds correlated with lower bipolar voltage measured by EAM [52]. The added value of this method is the possible standardization of the local scars. Currently, a threshold of 1.2 is commonly used to demarcate fibrosis [53]. Nevertheless, this ratio is highly dependent on multiple factors influencing the blood pool signal intensity, both technical (e.g., time of acquisition, quantity, and modality of contrast administered, scanner field strength, voxel size, image acquisition parameters, and time of inversion choice) and patient‐specific (e.g., contrast clearance rate and comorbidities).

Another method for the definition of fibrosis utilizes a threshold‐based SD above the mean signal intensity of normal myocardium or blood pool.

The Utah group proposed a score based on the scar extension, where atrial involvement is categorized as mild (when LGE is <15%), moderate (15%–35%), and extensive (>35%) [54]. Scar delineation is based on the assessment of higher signal intensity in the involved myocardium compared to the normal one. The DECAAF I study used a SD‐based method for evaluating the extension of LGE before the AF ablation. Results from this study show that the extent of LA LGE was associated with arrhythmia recurrence after the blanking period [55].

Hopman et al. compared a 3‐SD‐based and an IIR‐based methods for LA fibrosis quantification on two different software (CEMRG and ADAS 3D). The two software were used to measure fibrosis through IIR. Moreover, CEMRG was implemented to calculate fibrosis with the 3 SD method. To compare LA fibrosis quantification methods and alternative post‐processing software outputs, intraclass correlation coefficients were calculated. The LA fibrosis burden derived from the 1.2 threshold IIR was higher than that derived from 3 SD above the blood pool (29.80 ± 14.15% vs. 8.43 ± 5.42%; *p* < 0.001), to the point that one‐third of the patients were allocated to different fibrosis categories. Since these discrepancies might have clinical implications, further studies must be performed to verify which method has the best accuracy [56].

As per T1 and T2 mapping, few studies have investigated atrial T1 mapping and its possible role in the burden of AF. Beinart et al. outlined that an increase in post‐contrast T1 mapping values obtained from the posterior LA wall was associated with an elevation of voltages registered on EAM [57].

In addition to tissue characterization, LA function assessed by feature tracking‐CMR succeeded to stratify patients for AF recurrence after ablation. Lower baseline feature‐tracked LA strain, strain rate, and total LA emptying function measured before ablation were associated with higher AF recurrency [58]. In another study, baseline intra‐atrial dyssynchrony during sinus rhythm showed to be an independent predictor of recurrency [59]. Moreover, elevated LA sphericity index measured on CMR was linked to higher recurrence rate after AF ablation [60].

### 3.2 Intraprocedural

#### 3.2.1 LGE CMR Integration

Brisbal et al. conducted 15 reablations of pulmonary vein isolation (PVI) procedures guided by 3D‐LGE CMR reconstruction of the LA merged in the navigating system, with the operator blinded to electrical data on PV reconnections. These patients were compared to a control group of 15 patients re‐ablated with an octapolar circular catheter (Lasso, Biosense Webster) used to assess PV reconnections. The CMR‐guided procedure showed that it was able to identify atrial scarring and site of electrical reconnection of PVs, and feasible to guide ablation and PVs electrical isolation. Moreover, CMR‐guided re‐ablation showed shorter RF times, whereas total procedural times and fluoroscopy times did not differ [61]. Conversely, in the study by Quinto et al. involving 35 patients undergoing re‐PVI with a CMR‐guided procedure versus 35 re‐PVI patients undergoing the ablation of the PVI only, the CMR‐guided group had a shorter total procedure time and lower atrial arrhythmia recurrence rate at 24 months, suggesting that CMR guided procedure was more effective than the traditional one [62].

These promising results are questioned by larger studies: the DECAAF II and ALICIA trial. The DECAAF II trial compared patient undergoing ablation for persistent AF with a CMR guided procedure or with a traditional procedure (the ablation of the PVI only). Patients in the CMR guided procedure group received PV isolation and, after the entrance conduction block was confirmed, received ablation of fibrotic areas detected on CMR (either by encircling the fibrotic areas isolating them from the surrounding tissue or by “homogenization” of the fibrotic area through ablation lesions). These two techniques did not show any difference in terms of AF recurrence. Similarly, the ALICIA trial, did not show any difference in terms of AF recurrence rate between CMR‐guided ablation versus traditional procedure. Of note, the CMR guided group showed higher ablation‐related stroke events in the DECAAF II group while this was not observed in the ALICIA trial [62, 63].

#### 3.2.2 Real‐Time Visualization

As described for VT ablations, CMR can be performed prior to the procedure, atria can be segmented and a 3D shell can be reconstructed and merged with the EAM data [61, 63]. Moreover, real‐time CMR‐guided procedures have been studied in the last few years, either with PT or AT [25, 28].

**FIGURE 3:**
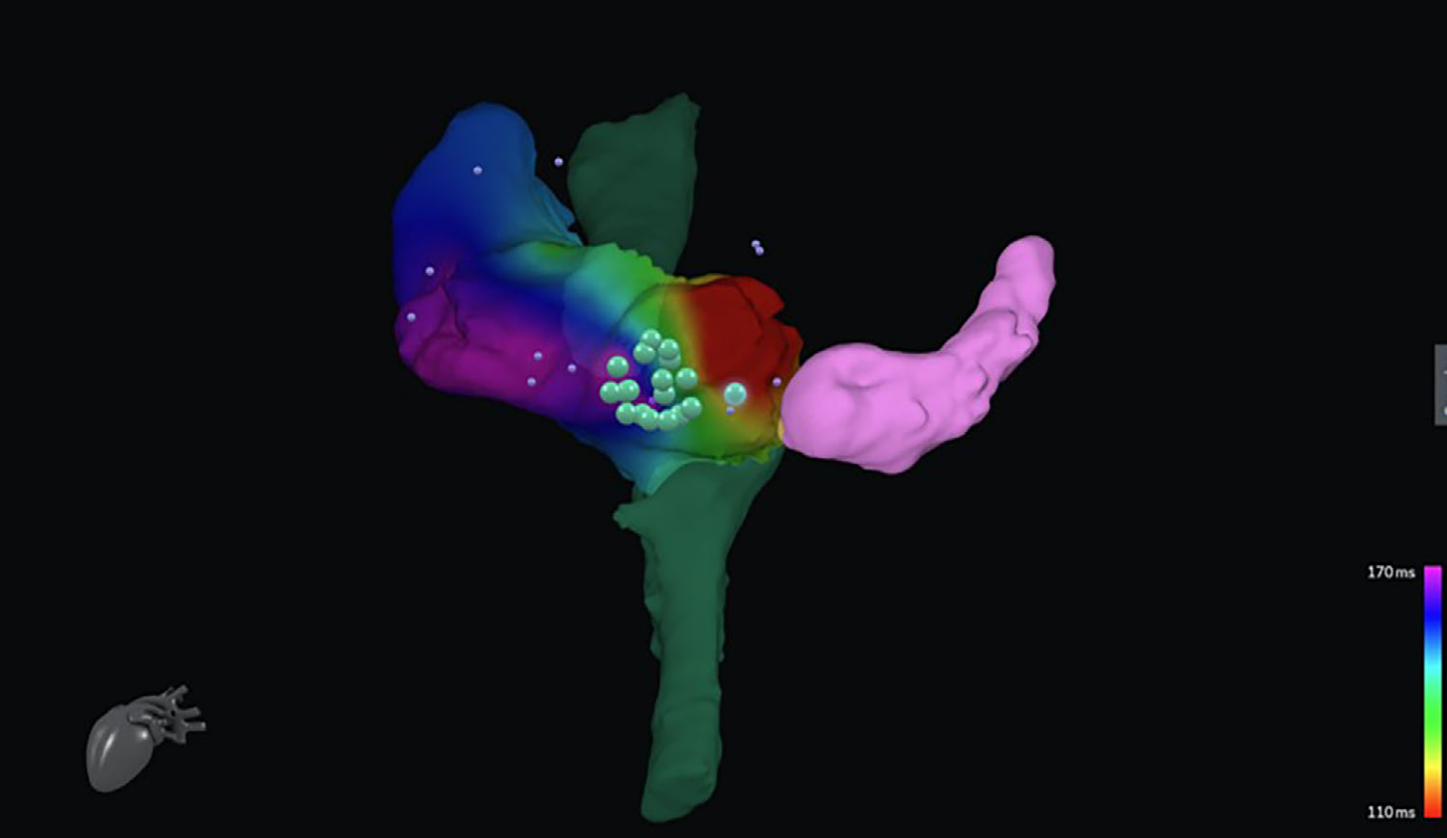
Real‐time CMR‐guided atrial flutter ablation. The use of a dedicated software allows for the merging of three‐dimensional anatomical reconstruction from MRI images and electrophysiological data. The inferior and superior vena cava are depicted in dark green, while coronary sinus in pink. An electro‐anatomical map is applied to show the activation pattern of the right atrium after the ablation. Big green spots represent the ablation points on the cavo‐tricuspid isthmus. Little pink spots represent mapping points.

Animal studies have proven the feasibility of CMR real‐time guidance at the level of the right atrium and right ventricle [64, 65]. Recently, CMR has been tested in human studies in patients with cavo tricuspid isthmus (CTI) dependent atrial flutter. Chubb et al. carried out fluoroscopy‐free ablation first on animals, then on 10 patients. The overall time of the procedure was longer (with an average of 5 h per procedure rather than a conventional mean time of 1 h when fluoroscopic guidance is used), and the medium‐term success rate was lower (72% vs. 85%–92% for conventional flutter ablation [25]. Similarly, Paetsch et al. performed atrial flutter ablation in 30 patients using pre‐procedural 3D LGE CMRs and AT without fluoroscopy. CMR‐guided atrial flutter ablation demonstrated to be feasible, with similar outcomes in terms of arrhythmia interruption and recurrence to conventional flutter ablations [66]. In addition, new vendor‐neutral software has been developed and showed to be feasible in RF ablation of flutter ablation (Figure 3) [67]. Although both studies stated that difficulties were initially encountered by the electrophysiologist performing the ablation, the potential reduction in radiation dose of the procedure plus the similar results in terms of recurrence rate suggest a major role for CMR in the future of atrial arrhythmia in those centers aiming to a zero‐ or near‐zero‐ionizing dose ablation [25, 66].

#### 3.2.3 Post‐Ablation

Atrial edema is also assessed with T2‐weighted sequences. Similar to ventricular locations, edema spreads outside of the actual ablation lesion, leading to overestimation of the ablation lesion with T2‐weighted images [68]. As interstitial edema expands the ECV, LGE in the acute settings overestimates the ablation lesion.

Native T1 sequences can be also used to identify atrial ablation lesions, such the novel long inversion time T1w (TWILITE) sequence and the T1 mapping. TWILITE has been validated in a swine study, showing it to be effective in identifying lesions in the acute setting as confirmed by a subsequent histological evaluation. The ablation leads to the oxidation of iron in myoglobin and hemoglobin. As the oxidated iron (ferric iron) is paramagnetic, T1 shortening occurs [69].

**FIGURE 4:**
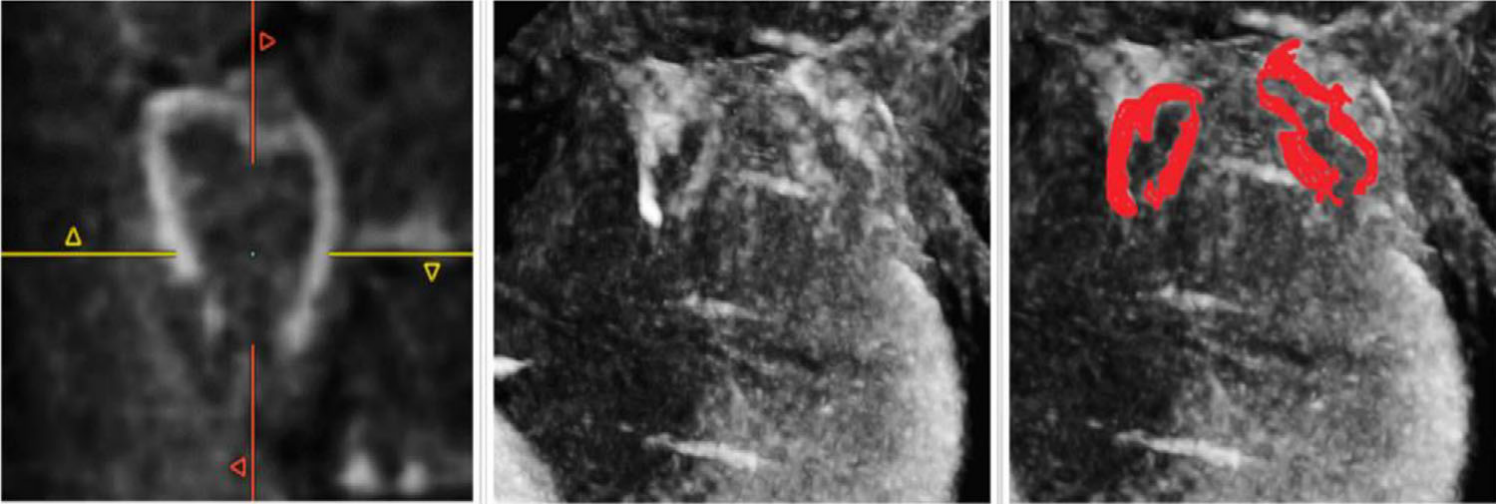
3D‐TWILITE images enhancing post‐ablation assessment of tissue damage after PV isolation. The 3D sequence allows for signal‐intensity‐based segmentation and reconstructions.

Guglielmo et al. demonstrated how TWILITE is more feasible than LGE in depicting the core of the lesions in a first human study, without the need for contrast agents. Nevertheless, TWILITE's ability to detect lesions was noticeably diminished when assessing posterior antral lesions [69, 70] (Figure 4).

**FIGURE 5:**
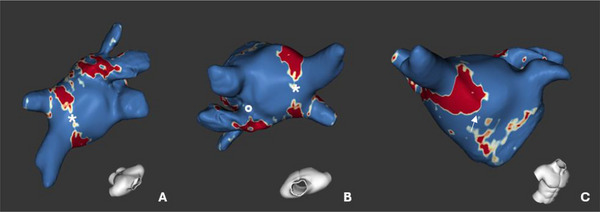
Assessment of chronic lesions and reconstruction with dedicated software (ADAS 3D LA, Galgo Medical, Barcelona, Spain). Red areas are the “core” zone reconstructed from areas with high signal intensity on 3D LGE sequences at CMR, yellow area of intermediate signal intensity, blue area of low signal intensity. (A) LA from a postero‐superior view, the left white * indicates a gap in the ablation of the roof of the left pulmonary veins where no LGE was found on CMR. (B) Superior view of the LA, white * marks the ablation lesion gap on the roof of the left pulmonary veins, and white ° indicates a gap area between the left upper PV and the LAA. (C) anterior view of the LAA, white arrow marks the encircling of the left pulmonary veins.

In a chronic setting, LGE CMR was able to predict durable PVI with very high positive predictive value (Figure 5) [71].

PFA is a novel, less invasive, nonthermal ablative that demonstrated to be feasible [72]. PFA induces cell lesion through electroporation resulting in cell death via multiple pathways: apoptosis, necrosis, necroptosis, or pyroptosis. In the acute setting, PFA lesions are characterized by interstitial edema while structural integrity remains mostly intacted [73]. These subtle alterations may contribute to reduced scar tissue formation compared to thermal ablation. Consequentially, the acute lesion size can be assessed with LGE, but since the ECV expansion is subtle, no lesion is appreciable in the chronic setting even with complete resolution of the arrhythmia [74, 75].

## 4 Conclusion

Treating arrhythmias remains a significant challenge. Despite advancements in new catheters and techniques, recurrence rates remain higher than desired [31]. In recent years, CMR has been increasingly integrated into the planning of ventricular arrhythmia ablations, allowing for precise identification of arrhythmogenic substrates. Additionally, the merging of 3D CMR‐derived anatomical shells with EAM data during ablation procedures has shown promising results [20]. In contrast, the role of CMR in the pre‐procedural planning of AF ablations is less well‐defined and CMR integration has been called into question by recent trials [53, 63].

In the acute post‐ablation setting, CMR is showing good results in depicting the ablation extension while in the chronic scenario it remains promising the possibility to target new ablation sites based on heterogeneity islets [13, 70]

Looking ahead, the development of CMR‐compatible catheters and dedicated sequences for catheter tracking shows exciting potential. One such application, so far limited to CTI‐dependent atrial flutter ablation, is the real‐time guidance of electrophysiological procedures, which could offer valuable advancements in arrhythmia treatment.
